# Hepatitis B virus genotypes and precore and core mutants in UAE patients

**DOI:** 10.1186/1743-422X-7-160

**Published:** 2010-07-15

**Authors:** Mubarak Alfaresi, Abida Elkoush, Hajer Alshehhi, Azza Alzaabi, Adeel Islam

**Affiliations:** 1 Department of Pathology &Laboratory Medicine, Zayed Military Hospital, Abu Dhabi, UAE

## Abstract

**Background:**

Knowledge of the HBV genotype with which a patient is infected is crucial information for a physician to have when planning clinical treatment for that patient. Previous studies have suggested that there are possible differences in the pathogenicity and therapeutic response of different HBV genotypes. However, the prevalence of the various HBV genotypes and Precore and Core mutations is unknown in the UAE. Therefore, we sought to determine the prevalence of the different HBV genotypes in the UAE population.

**Methodology/Principal Findings:**

A total of 88 HBsAg-positive patients were included in the study.

A method for genotyping and subtyping HBV by partial HBsAg gene sequencing using primers that are complementary to all known genotypes was used. Precore and core region of these viruses were also sequenced in 88 patients.

HBV genotype D was the most prevalent (79.5%) genotype identified in our study population, followed by genotypes A (18.2%) and C (2.3%). The following subtypes were isolated: ayw2 (80.7%), adw2 (14.8%), and adw (2.3%). The HBV-DNA viral load was higher in HBeAg-positive patients than it was in patients who were HBeAg-negative. Precore mutants were found in 51 (58.0%) of 88 patients. Mutations in the basal core promotor were found in 22 (25.3%) of 88 patients.

**Conclusion/Significance:**

HBV infection is a major health problem in the UAE, and while genotypes B and C are the most prevalent HBV genotypes in the Asian population, our study reveals that genotype D is the predominant genotype that is present in the UAE. More patients were HBeAg-negative than were HBeAg-positive in our study sample, which could be due to the duration of infection of the included patients. Additionally, the viral loads of the HBeAg-positive patients were higher those of the HBeAg-negative patients. Analysis of nucleotide 1858 showed presence of thymine in all patients with genotypes C, and D and in a few patients with genotypes A. This nucleotide was closely related to the presence of precore mutants. Mutations in the basal core promoter were found in 22 of 88 (25.3%) samples. These mutations were more frequent in patients infected with genotype A (37.5%) and not found in patients infected with genotype C.

## Background

Hepatitis B Virus (HBV) is a well-known agent of acute and chronic hepatitis, liver cirrhosis and hepatocellular carcinoma. Around 400 million people worldwide carry the virus of which more than 250 million reside in Asia[1].

The course of the disease can vary from a subclinical self-limited illness to chronic active hepatitis, which can either lead to death after many years or to fulminant hepatitis[2]. The chronic carrier state occurs in 5 to 10% of individuals who are infected as adults and in 85 to 90% of those who are infected during infancy[3]. The outcome of infection depends upon many factors, such as the host immune status, their age at the time of infection, and the degree of viral replication that occurs. Another factor that has been postulated to affect the outcome of infection is the genetic variability of the virus, which influences its expression of viral antigens[4]. However, the impact that the natural genetic variability of the virus has on infected patients' clinical course has only recently become a topic of research.

HBV was formerly classified into four different subtypes that were afterward subdivided according to the antigenic determinants of HBsAg in adw (adw2 and adw4), ayw (ayw1, ayw2, ayw3, and ayw4), adr (adrq adrq), and ayr. Subtype a is common to the majority of viruses and is related to a neutralizing epitope. Divergence of the complete genome in a same subtype is ca. 8%, similar to the one found between different subtypes[5].

Genotypically, HBV is divided into eight groups, A-H. These groups were identified based on an intergroup divergence of 8%[5] or 4% in the gene S sequence[6]. Genotype A is pandemic and is most prevalent in Northern Europe, North America, and Central Africa. Isolates of genotypes B and C have been observed in Southeast Asia and the Far East. Genotype D is distributed worldwide and is most prevalent in the Mediterranean region. Genotypes E and F are prevalent in West Africa and in the Amerindian population, respectively[7,8]. Recently, genotype G was identified in the USA and France[9]. Genotype H was also recently found in Central America[10]. The genotypes and subtypes are useful clinical and epidemiological markers[11,12] because it is well known that genotypes vary geographically and correlate strongly with ethnicity[4,7].

In the natural course of chronic HBV infection, the loss of HBeAg expression and the appearance of antibodies directed against it (Anti-HBe) are usually accompanied by cessation of viral replication. However such a serology profile may also be seen in individuals who harbor pre- core (PC) and basal core promoter (BCP) mutants where replicative infection continues. The frequent genomic mutation that leads to HBeAg negativity is the mutation of the nucleotide (nt) 1896 from G to A (G-A). This mutation converts codon 28 of the precore sequence to a termination codon (TGG→TAG) and thus prevents HBeAg from being expressed[13]. PC variants are more common among patients with genotype D (65 to 75 percent) than genotype A (9 to 18 percent)[14,15].

A second group of mutations affect the basal core promoter region and result in a transcriptional reduction of precore but not pregenomic and core mRNA[16]. These HBeAg suppressive strains contain mutations of nt1762 from A to T (A1762T) and nt 1764 from G to A (G1764 A) in the BCP region and are the predominant quasispecies in chronic hepatitis patients[17-20]. These mutations may be found in isolation or in conjunction with PC mutations. Occurrence of these mutations result in increase in viral load[16,17,21,22]. These changes were initially thought to be related to a "HBeAg-negative phenotype" but recent studies showed that they may also be found in some HBeAg-positive patients, especially those with chronic hepatitis[18,23].

In the present study, we sought to determine the prevalence of the various HBV genotypes in the UAE as this information was previously unknown. We determined the HBV genotype, subtype, viral load, and HBeAg antibody status and examined those and other clinical characteristics (including age and gender) of the patients included in our study to determine if there was a correlation between the molecular characteristics of the HBV virus with which a patient was infected and their clinical characteristics. We also verified the frequency of precore and BCP mutations in the UAE patients.

## Methods

### Patients

A total of 88 consecutive serum samples from HBsAg-positive patients between the period of January 2008 till December 2009 were evaluated in this study. Most of samples collection was done with no relation of symptoms appearance, but as a routine check up or for follow up. These samples were derived from 74 males and 14 females with a mean age of 35.33 ± 11.5 years (range: 18 to 70 years). All of these patients were UAE citizens. Serum samples were stored at -20°C and thawed immediately before use. This work has been approved by the Zayed military hospital. No written consent was needed for this work since no additional sample was taken for the study. The samples were evaluated for the presence of several serological markers of HBV infection (including HBeAg, anti-HBeAg, and HBsAg) using the bioMérieux ELISA kit according to the manufacturer's instructions.

### Detection of HBV-DNA by PCR (polymerase chain reaction)

The extraction and amplification of HBV-DNA was carried out by nested PCR using the methods described by Kaneko et al.[24].

### Analysis of HBV sequences from different genotypes

We used selected primers that have been described previously[25] and that corresponded to conserved regions of the various HBV genotypes that flank heterogeneous intervening regions to distinguish between the HBV genotypes. The region selected for amplification also included the amino acid loop corresponding to the a, d/y, and w/r allelic subtypic determinants as well as mutations that have been shown to be related to the HBIg antibody, the anti-HBs monoclonal antibody, and vaccine resistance. The following primers were selected: 1) FHBS1, 5'-GAG TCT AGA CTC GTG GTG GAC TTC-3'; 2) FHBS2, 5'-CGT GGT GGA CTT CTC TCA ATT TTC-3'; 3) RHBS1, 5'-AAA TKG CAC TAG TAA ACT GAG CCA-3'; and 4) RHBS2, 5'-GCC ARG AGA AAC GGR CTG AGG CCC-3'. The positions in the HBV genome (strain HBVADW; GenBank accession number V00866) to which the primers corresponded were as follows: 1) HBS1F (positions 244 to 267), 2) HBS2F (positions 255 to 278), 3) HBS2R (positions 648 to 671), and 4) HBS1R (positions 668 to 691). Serum samples were treated as described above and subjected to two rounds of amplification sequentially with outer (FHBS1 and RHBS1) and inner (FHBS2 and RHBS2) primers. The amplification conditions for the two rounds of the nested PCR were as follows: initial denaturation at 94°C for 20 s, followed by 30 cycles of amplification at 94°C for 20 s, 56°C for 20 s, and 72°C for 30 s, followed by a final extension step at 72°C for 1 min in a PTC-200 Thermocycler (MJ Research, Watertown, Mass.).

### Detection of BCP and precore mutants

For the detection of BCP and precore mutants, HBV-DNA-positive samples were amplified by using the primers described by Takahashi et al.[19].

### Sequencing reaction

PCR products were subjected to cycle sequencing reactions as described previously[26] using the second round primers and the ABI Prism BigDye Terminator Cycle Sequencing Ready Reaction Kit (Applied Biosystems, Foster City, Calif.). After purification, the samples were denatured and loaded onto a 5% Long Ranger 6 M urea gel (Long Ranger Gel Solution; FMC) and sequenced using an automated ABI Prism 377 DNA Sequencer (Applied Biosystems).

### Sequence analysis

Genotyping, BCP, and precore mutant analysis were carried out by sequence comparison with known sequences from different HBV genotypes that have been previously described and were aligned as described above. The Geneious program (Biomatters, Inc.) was used for genotyping as well as for phylogenetic and molecular evolutionary analyses. The DNA sequences obtained from the PCR analyses were aligned using Geneious and then analyzed using the neighbor-joining method via a distance matrix that was calculated using the Kimura two-parameter model[24]. Woolly monkey hepatitis B virus (GenBank accession number AF046996) was used as an outgroup.

### HBV DNA Quantification

All samples were submitted to HBV DNA quantification using the commercial TaqMan Amplicor HBV assay (Roche Diagnostics), which has a lower limit of detection of 12 IU/L.

### Statistical analysis

For statistical analysis, we used the PASW Statistics software package, version 18.0. Either the χ^2 ^test with the Yates correction or Fischer's exact test was used to analyze quantitative data and to compare proportions. All calculated P-values were two-tailed and all P-values < 0.05 were considered to be statistically significant.

### GenBank accession numbers

Sequences from the S gene that were acquired during this study were deposited in the GenBank under numbers GU594063-GU594150.

## Results

**Figure 1:**
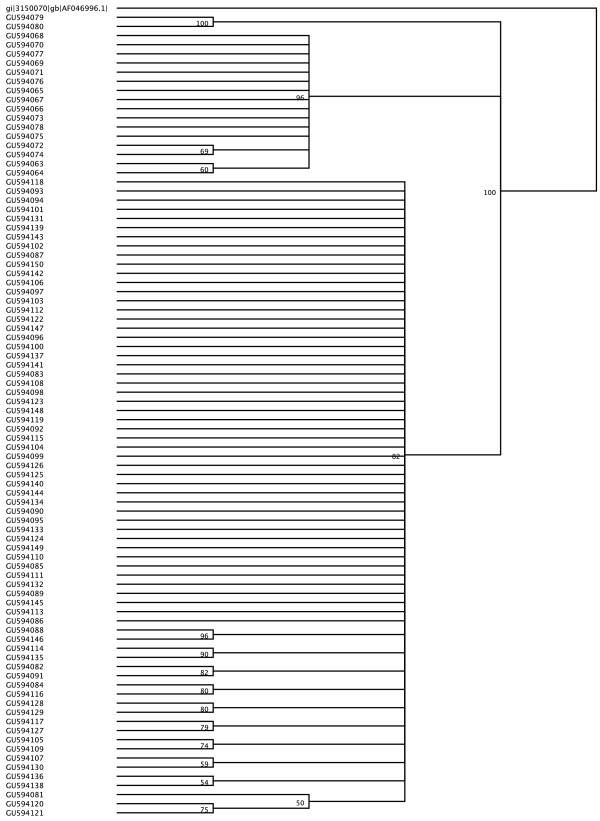
**Neighbor-joining tree of a specific HBV-DNA nucleotide fragment (S gene) from viral isolates obtained from the study population. Woolly monkey Hepatitis B virus (GenBank accession number **AF046996**) was used as an outgroup**.

The baseline characteristics of the study population as well as the frequencies with which the various HBV genotypes and subtypes were observed are shown in Table 1. All 88 patients (100%) were citizens of the UAE. In this group [as was shown in phylogenetic analysis (Fig. 1)], genotype D was the most prevalent (79.5%) followed by genotype A (18.2%) and genotype C (2.3%). The following subtypes were detected: *ayw2 *(80.7%), adw2 (14.8%), and *adw *(2.3%). Two patients infected with HBV genotype C could not be subtyped. Within genotype A, subtypes *adw *and *adw2 *were detected, while within genotype D, only subtype *ayw2 *was detected.

**Table 1:** Baseline characteristics and HBV genotype frequencies of the 88 HBV-infected patients included in this study

HBeAg-positive patients		N = 88 (%)
Gender	M/F	74/14(84/16)
Mean age	Years	35.33 ± 11.5
Age range	Years	18-70
		
Genotype	A	16 (18.2)
	C	2 (2.3)
	D	70 (79.5)
		
Subtype	ayw2	71 (80.7)
	adw2	13 (14.8)
	adw	2 (2.3)
		
HBeAg status	Positive	5 (5.7)
		
Inactive carrier	ALT < 65 U/L	71 (80.7)
Chronic hepatitis	ALT ≥65 U/L	17 (19.3)

The prevalence of each of the HBV genotypes that were isolated from the included patients was also assessed with respect to patient age (Table 2). There was no trend observed in the distribution of genotypes among the various age groups (P = 0.674). However, genotype A and C were not observed in individuals aged between 10 and 20 years of age, whereas genotype D was observed in all age groups. Males comprised a larger proportion of our study population than females, and because our study population was comprised of 88 consecutive HBV-infected patients, males appeared to be more frequently infected with HBV than females.

**Table 2:** Distribution of HBV genotypes by patient age

Age group (years)	Genotype A (N)	Genotype C (N)	Genotype D (N)
1 to 20	0	0	5
21 to 40	12	1	43
41 to 60	3	1	21
> 60	1	0	1

Total	16	2	70

**Figure 2:**
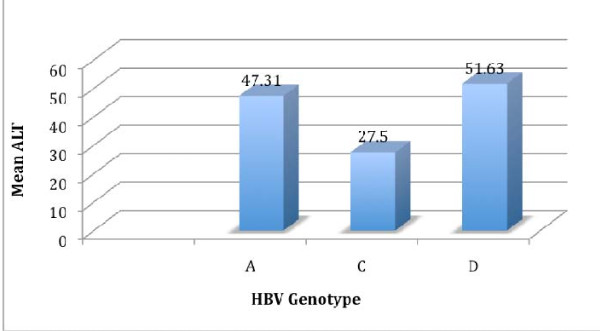
**Mean ALT values(U/L) of patients infected with the three HBV genotypes that were identified in this patient population**.

We compared the mean alanine aminotransferase (ALT) levels of patients infected with each of the three genotypes that were detected. The highest mean ALT level (51.30 ± 18.32 U/L) was found in the individuals infected with HBV genotype D (Fig. 2). However, there was no significant difference in ALT level observed with respect to HBV genotype (P = 0.27).

In our study, 83 (94.3%) of patients were HBeAg-negative and 5 (5.7%) were HBeAg-positive (Table 3). We did not observe any statically significant differences between HBeAg-negative and HBeAg-positive patients with regard to gender, age, or HBV genotype. The mean HBV-DNA level was significantly higher among HBeAg-positive patients (3.02 × 10^7 ^IU/mL) than it was in HBeAg-negative patients (3205964 IU/mL; P = 0.004). Additionally, HBeAg-positive patients were more likely to have chronic hepatitis than HBeAg-negative patients (P = 0.048).

**Table 3:** Baseline characteristics and genotype distribution frequencies of the included HBV-infected patients, stratified by HBeAg status (n = 88)

Characteristics		HBeAg-positive	HBeAg-negative	P-value
		(n = 5)	(n = 83)	
Mean age	Years	39 ± 10.2	35 ± 11.6	0.377
				
Gender	M	3 (60%)	71 (85.5%)	
	F	2 (40%)	12 (14.5%)	0.178
				
Genotypes	A	1 (20%)	15 (18%)	0.13
	C	0	2 (2.4%)	
	D	4 (80%)	66 (79.6%)	
				
Inactive carrier	ALT < 65 U/L	2 (40%)	69 (83%)	0.048
Chronic hepatitis	ALT ≥65 U/L	3 (60%)	14 (17%)	
				
HBV-DNA viral load	(IU/mL)	3.02 × 10^7^	3205964	0.004

In 88 patients, we also analyzed the BCP and precore region of HBV. The frequency of mutants in these regions among the different genotypes, as well as the results of the nt 1858 analysis, are shown in Table 4. Precore mutants were more frequent in patients infected with genotype C and D virus (P < 0.0001).

**Table 4:** HBV genotypes and their relationship to viral features

HBV		BCP and precore region mutations(no. [%])				
	
genotype	B	CP	nt	1858	Precore	region
			
	WT	Mutant	C	T	WT	Mutant
A	10(62.5)	6(37.5)	7(43.8)	9(56.3)	15(93.8)	1(6.3)
C	2(100)	0	0	2(100)	0	2(100)
D	53(76.8)	16(25.3)	0	70(100)	22(31.4)	48(68.6)
						
Total	65(74.7)	22(25.3)	7(8)	81(92)	37(42)	51(58)

Analysis of nt 1858 showed the presence of thymine in all patients with genotypes C and D and 56.5% in patients with genotype A. This nucleotide was closely related to the presence of precore mutants (P = 0.002).

Mutations in the basal core promoter were found in 22 of 88 (25.3%) samples. These mutations were more frequent in patients infected with genotype A (37.5%), less frequent among genotype D-infected patients (23.2%) and not found in patients infected with genotype C, but no statistical significant difference was found.

## Discussion

HBV infection is an important global health problem that places a continuously increasing burden on developing countries like the UAE. About 400 million people worldwide are chronic carriers of the virus[1]. In addition to the serological classification of HBV isolates into nine subtypes on the basis of HBsAg determinants[27], a genetic classification based on the comparison of complete genomes has defined eight genotypes of HBV (A to H).

Differences in the distribution and clinical characteristics of the eight HBV genotypes have been studied extensively around the world. Better responses to treatment have been reported for genotypes A and B than genotypes D and C[28-30]. On the other hand, progression to chronic hepatitis or to more severe diseases, such as hepatocellular carcinoma, has been shown to occur most frequently in patients infected with genotypes A and C[28,31-33]. Data regarding genotype F that has been reported thus far have been scant, but in one study, death related to liver disease was observed more frequently in patients infected with genotype F than in those infected with genotypes A or D[29].

The 88 samples that were genotyped indicated that genotype D had the highest prevalence in our population, followed by genotype A. Genotypes E and F were not isolated from any of our patients, indicating that these genotypes are not present in this region. Interestingly, the initial studies on HBV genotype distribution in other parts of Asia found that genotypes B and C were the most prevalent genotypes in this region. However, almost all of these studies were performed in Japan and China, which are geographically distant from the UAE. Later studies revealed that seven of the eight HBV genotypes are present in Asia[34]. For instance, the predominant HBV genotypes in India have been shown to be genotypes A and D[35], while the predominant HBV genotype in Afghanistan was found to be genotype D[36]. The epidemiological data about the prevalence of the seven HBV genotypes that have been observed in Asia have revealed the predominance of genotype D in this region.

Genotypes E, G, and H were not found in our study population. Genotype E has only been isolated in certain regions of Africa[37] and in one Haitian child who was infected with this HBV genotype in Belgium[38]. Genotype G was found to be present in about 10% of patients in France and United States in a previous epidemiological study[9].

In this study, the most important predictor of an elevated ALT level and a high HBV-DNA level was HBeAg status, as HBeAg-positive patients were more likely to have higher ALT levels and HBV-DNA levels than HBeAg-negative patients.

Among the HBeAg-positive patients, we found that 40% were inactive carriers, compared to 60% of patients with chronic hepatitis B (Table 3). Among 88 patients included in this study, all of whom were HBsAg-positive and HBV-DNA positive, 5 (5.7%) were HBeAg-positive and 83 (94.3%) were HBeAg-negative. Therefore, in our population, a high percentage of HBV appear to be HBeAg-negative. In other studies that have been performed in different patient populations, considerable differences between the percentages of HBeAg-positive and HBeAg-negative patients were also observed. These previous authors encountered a higher prevalence of HBeAg-negative patients than HBeAg-positive patients in their studies, with HBeAg negativity rates varying from 52.5% to 63.3%[39-41]. The majority of the HBV-positive patients in our study had probably been infected for a long time and had therefore likely developed mutations in the pre-core region. Therefore, a number of included patients were probably HBeAg-negative but anti-HBeAg-positive.

Precore mutants had an intermediate frequency in our population (58%). Such mutants were found in all patients infected with genotype C, with high frequency in patients infected with genotypes D, and at a very low frequency in genotype A-infected patients. The occurrence of this mutation is dependent upon the nucleotide (cytosine or thymine) at position 1858, which forms a base pair with nt 1896 in the pregenomic RNA loop at the ε encapsidation sign. A thymine at position 1858 is particularly common in genotype D viruses. The presence of a cytosine at position 1858 precludes the G-to-A mutation at nt 1896, since this would destabilize the stem-loop structure of the RNA encapsidation signal[13]. Interactions of this encapsidation signal with the viral DNA polymerase is an essential step in the viral replication cycle, and it has been hypothesized that the increased strength of the guanine-to-cytosine base pairing found in pre-core mutants would implicate in a stronger ε encapsidation sign, allowing a more efficient replication of these strains. Genotype A usually shows a cytosine at this position[42]. Our results from nt 1858 analysis corroborate with these data, since we also have found the presence of cytosine only in genotypes A, which showed a low frequency of precore mutants.

The frequency of this mutation varies widely around the world. Castro et al.[43] studied Brazilian patients and found the precore stop codon mutation in only 24% of them--a result similar to ours. These authors also demonstrated the higher frequency of the stop codon mutation at nt 1896 in the isolates that had T1858. On the other hand, the prevalence of this mutation in China was 86%[44]. This discrepancy may reflect differences in genotype distributions in each studied population, since genotype D viruses are common in the UAE populations, whereas in China genotypes B and C are more common.

The BCP mutation was found in 25.3% of our patients and was present in A and D genotypes. BCP mutants frequency ranged from 23.2% in genotype D to 37.5% in genotype A. On the other hand, other authors have reported that the presence of these mutants is not related to the presence of precore mutants[43]. This point should be further analyzed in our population.

## Conclusion

HBV genotype influences the severity of liver disease that patients experience as well as their response to interferon and antiviral therapy. It is also thought to influence the emergence of resistant strains. Therefore, patients infected with certain genotypes of HBV that are known to be resistant to common treatment regimens can be counseled to seek alternative therapeutic options to spare them the cost and burden of treatment. The knowledge of the prevalence of HBV genotypes in a certain region is thus of immense importance to allow the proper and effective management of HBV patients in that region. As we have reported for the first time, HBV genotype D appears to be the predominant genotype in the UAE. In this study, the viral loads of HBeAg-positive patients were higher than those of HBeAg-negative individuals. Therefore, it should be considered worthwhile for clinicians to adopt better strategies to prevent and cure HBV infection. Precore mutants are more common among genotype C and D-infected patients, whereas BCP mutants were present in A and D genotypes. These types of studies are thus important both for epidemiological reasons and because they can help promote efforts to develop effective treatments for HBV.

## Competing interests

The authors declare that they have no competing interests.

## Authors' contributions

MS: conceived and designed the experiments, participated in experiments, analyzed data, wrote the paper, AE: participated in experiments, HA; participated in experiments, AA: participated in experiments, AI: contributed to data analysis.

All authors have read and approved the final manuscript.
